# The Endothelial Dysfunction Could Be a Cause of Heart Failure with Preserved Ejection Fraction Development in a Rat Model

**DOI:** 10.1155/2022/7377877

**Published:** 2022-05-18

**Authors:** Thomas Dupas, Thomas Pelé, Justine Dhot, Mélanie Burban, Antoine Persello, Virginie Aillerie, Angélique Erraud, Angela Tesse, David Stevant, Angélique Blangy-Letheule, Céline Menguy, Vincent Sauzeau, Michel De Waard, Bertrand Rozec, Chantal Gauthier, Benjamin Lauzier

**Affiliations:** Nantes Université, CHU Nantes, CNRS, INSERM, l'institut du thorax, F-44000 Nantes, France

## Abstract

50% of patients with heart failure have a preserved ejection fraction (HFpEF). Numerous studies have investigated the pathophysiological mechanisms of HFpEF and have shown that endothelial dysfunction plays an important role in HFpEF. Yet no studies answered whether endothelial dysfunction could be the cause or is the consequence of HFpEF. Recently, we have shown that the endothelial overexpression of human *β*_3_-adrenoreceptor (Tg*β*_3_) in rats leads to the slow development of diastolic dysfunction over ageing. The aim of the study is to decipher the involvement of endothelial dysfunction in the HFpEF development. For that, we investigated endothelial and cardiac function in 15-, 30-, and 45-week-old wild-type (WT) and Tg*β*_3_ rats. The aortic expression of ^**•**^NO synthase (NOS) isoforms was evaluated by Western blot. Finally, electron paramagnetic resonance measurements were performed on aortas to evaluate ^**•**^NO and O_2_^•-^ production. Vascular reactivity was altered as early as 15 weeks of age in response to isoproterenol in Tg*β*_3_ aortas and mesenteric arteries. NOS1 (neuronal NOS) expression was higher in the Tg*β*_3_ aorta at 30 and 45 weeks of age (30 weeks: WT: 1.00 ± 0.21; Tg*β*_3_: 6.08 ± 2.30; 45 weeks: WT: 1.00 ± 0.12; Tg*β*_3_: 1.55 ± 0.17; *p* < 0.05). Interestingly, the endothelial NOS (NOS3) monomer form is increased in Tg*β*_3_ rats at 45 weeks of age (ratio NOS3 dimer/NOS3 monomer; WT: 1.00 ± 0.37; Tg*β*_3_: 0.13 ± 0.05; *p* < 0.05). Aortic ^•^NO production was increased by NOS2 (inducible NOS) at 15 weeks of age in Tg*β*_3_ rats (+52% vs. WT). Aortic O_2_^•-^ production was increased in Tg*β*_3_ rats at 30 and 45 weeks of age (+75% and+76%, respectively, vs. WT, *p* < 0.05). We have shown that endothelial dysfunction and oxidative stress are present as early as 15 weeks of age and therefore conclude that endothelial dysfunction could be a cause of HFpEF development.

## 1. Introduction

Over the past decade, cardiovascular diseases have been one of the leading causes of death worldwide [1]. Among these diseases, heart failure (HF) affects 2-3% of the world's population [2]. There are several forms of HF, including HF with preserved ejection fraction (HFpEF) which has no efficient treatment at present day. This type of HF is characterized by a diastolic dysfunction without major alteration of ejection fraction. The main clinical characteristics of patients with HFpEF are advanced age and female sex [3]. However, the mechanisms leading to HFpEF are not completely understood so far. Few years ago, Paulus and Tschöpe put forward the idea that endothelial function could be the common trigger of all HFpEF etiologies. More specifically, the nitric oxide (^•^NO) pathway was pointed out [4]. Indeed, the ^•^NO is a major player in the maintenance of cardiovascular function. The ^•^NO is mainly produced by the endothelial cells *via* the activation of the ^•^NO synthase (NOS) which exists under three isoforms: the neuronal NOS (nNOS or NOS1), the inducible NOS (iNOS or NOS2), and the endothelial NOS (eNOS or NOS3). In physiological conditions, most of the ^•^NO production in the vessels and the heart is mediated by NOS3 [5]. Paracrine action of ^•^NO, produced by endothelial cells, induces a relaxation of smooth muscle cells in the vessels. At the cardiac level, endothelial cells represent 20% of the cellular population and the ^•^NO plays a role in cardiac contractility regulation [6]. The ^•^NO production is partly mediated by the activation of *β*-adrenoceptors (*β*-AR) and more specifically the *β*_3_-adrenoceptor (*β*_3_-AR) through the activation of NOS3 or NOS1 [7, 8]. *β*_3_-AR activation, expressed in cardiomyocytes and endothelial cells, induced ^•^NO release [9]. Considering the putative link between HFpEF and endothelial dysfunction and the link between *β*_3_-AR and ^•^NO signaling, the purpose of the study is to evaluate the link between endothelial dysfunction and HFpEF development. An animal model that overexpresses the human *β*_3_-AR (Tg*β*_3_) on endothelial cells has been developed. Previously, we have shown that Tg*β*_3_ animals overproduced ^•^NO at the cardiac level and develop a diastolic dysfunction at 45 weeks of age, characteristic of HFpEF [10]. The aim of this study is to understand if endothelial dysfunction could be the cause of HFpEF.

## 2. Methods

### 2.1. Experimental Animals

All animal experimental protocols were approved by the Pays de la Loire Ethical Committee and were performed in accordance with the French law on animal welfare, EU Directive 2010/63/EU for animal experiments, the National Institutes of Health (NIH) Guide for the Care and Use of Laboratory Animals (NIH Pub. No. 85-23, revised 2011), and the 1964 Declaration of Helsinki and its later amendments, and all the animals were housed according to standard living conditions. All animals used in the study are male Sprague-Dawley rats. Tg*β*_3_ rats which overexpress human *β*_3_-AR in endothelial cells and their control (WT) were used at 15, 30, and 45 weeks of age and fed *ad libitum* with a soy-free diet (Envigo, #2914C, Huntingdon, United Kingdom) as previously described [10]. Then, animals were euthanized, and the thoracic aorta and superior branch of mesenteric arteries were harvested in order to perform vascular reactivity (thoracic aorta and mesenteric arteries), electronic paramagnetic resonance (EPR) quantification of ^•^NO and O_2_^•-^ (thoracic aorta), and Western blot protein analysis (thoracic aorta) as described below.

### 2.2. Echocardiography

Echocardiography was performed on anesthetized rats (O_2_/isoflurane mixture 1.5% with flow rate of 1 L/min) at 15, 30, and 45 weeks of age, using a Vingmed-General Electric ultrasound system (VIVID 7, Horten, Norway) equipped with a 10 MHz imaging probe and offline cine loop analysis software (Echopac TVI, GE-Vingmed Ultrasound) on the Therassay platform of Nantes, as previously described [10].

### 2.3. Vascular Reactivity for Pharmacological Studies

Rats were anesthetized with an O_2_/isoflurane mixture (induction: 5% isoflurane, flow rate 1 L/min; maintenance: 2% isoflurane, flow rate 0.5 L/min). The thoracic aorta and mesenteric arteries were carefully excised and cleared of fat and connective tissue. Vascular tensions were recorded as previously described [11, 12]. Briefly, aortic and mesenteric rings were mounted on a multichannel isometric myograph (Danish Myo Technology) (95% O_2_ and 5% CO_2_) Krebs-Henseleit bicarbonate solution (millimolar concentrations: NaCl 118, NaHCO_3_ 25, KH_2_PO_4_ 1.2, MgSO_4_ 1.2, KCl 4.5, glucose 11, and CaCl_2_ 1.5) at 37°C [13]. To verify the endothelium integrity, rings were precontracted with phenylephrine (1 *μ*M, Sigma-Aldrich, P6126) and then exposed to a single concentration of acetylcholine (10 *μ*M, Sigma-Aldrich, A6625). The tension was recorded on precontracted rings with phenylephrine (1 *μ*M). The vasodilation response to cumulative concentrations of isoproterenol, a nonselective *β*-AR agonist (1 nM to 100 *μ*M, Sigma-Aldrich, I6504), and of CL 316 243, a *β*_3_-AR agonist (1 nM to 100 *μ*M, Tocris, 1499), was evaluated. The vasodilation response to isoproterenol (1 nM to 100 *μ*M) was repeated after 30 min of vessel incubation with NOS inhibitors prior to the phenylephrine precontraction: L-NIO, a NOS3 inhibitor (10 *μ*M, *N*^5^-(1-iminoethyl)-L-ornithine dihydrochloride, Tocris, 0546); vinyl-L-VNIO, a NOS1 inhibitor (10 *μ*M, N^5^-(1-imino-3-butenyl)-L-ornithine, L-VNIO, Enzo Life Sciences, ALX-270-216-M005); 1400W, a NOS2 inhibitor (10 *μ*M, *N*-[[3-(aminomethyl)phenyl]methyl]-ethanimidamide dihydrochloride, Tocris, 1415); and L-NMMA, a nonselective NOS inhibitor (30 *μ*M, NG-monomethyl-L-arginine acetate, Tocris, 0771) were evaluated. A chamber wire myograph was connected to a digital data recorder (MacLab/4e; AD Instruments), and recordings were analyzed using LabChart v7 software (AD Instruments).

### 2.4. Electronic Paramagnetic Resonance (EPR)

EPR measurements were performed on aortas harvested from rats at 15, 30, and 45 weeks of age, as previously described [14]. Briefly, aortas were incubated 45 min at 37°C in a Krebs-HEPES colloid solution containing Na-diethyldithiocarbamate trihydrate (DETC—Sigma-Aldrich) mixed to FeSO_4_-7H_2_O to form Fe^2+^-(DETC)_2_ as spin trap for ^•^NO detection in an electromagnetic field. Five conditions were tested: without inhibitors, with L-NMMA (10 *μ*M, negative control), with L-NIO (10 *μ*M) and L-VNIO (10 *μ*M), with L-NIO (10 *μ*M) and 1400W (10 *μ*M), and with L-VNIO (10 *μ*M) and 1400W (10 *μ*M). For O_2_^•-^ detection, aortas were incubated 45 min at 37°C in a Krebs-HEPES solution containing 1-hydroxy-3methoxycarbonyl-2,2,5,5-tetramethylpyrrolidin (CMH, 500 *μ*M, Noxygen) as spin probe, deferoxamine (25 *μ*M, Sigma-Aldrich) as an iron chelator, and DETC (5 *μ*M) to minimize CMH autooxidation, with or without L-NMMA (10 *μ*M). Then, each sample was snap-frozen in liquid nitrogen and analyzed in a Dewar flask at 77°K using an EPR Miniscope MS5000 (Freiberg Instruments, Germany). The instrument settings were as follows: microwave power of 10 mW; 1 mT or 0.400 mT of amplitude modulation for ^•^NO and O_2_^•-^, respectively; 100 kHz modulation frequency; sweep time of 150 s; and 3 scans for ^•^NO measurements or 60 s and 3 scans for O_2_^•-^ spectra. Signals were quantified by measuring and analyzing the total amplitude of the peaks of the spectra obtained, using the ESRS-Studio software (Freiberg Instruments, Germany) and expressed in arbitrary units (A.U.) and normalized to dry weight of the sample.

### 2.5. Western Blot

Total proteins were extracted from aorta powder as previously described [15]. The amount of protein used for Western blot analysis was 25 *μ*g. Western blots were performed with a migration in 4-15% polyacrylamide stain-free gels in order to evaluate the expression of the NOS1 (#4231S, Cell Signaling, Danvers, USA), NOS2 (AB5382, Millipore, USA), NOS3 (610296, BD Biosciences, San Diego, USA), and phosphorylated Ser1177 NOS3 (p-NOS3; #9571, Cell Signaling, Danvers, USA) (Table 1). Total NOS1, NOS2, and NOS3 expression was expressed as a ratio with stain-free quantification of total proteins. Phosphorylated Ser1177 NOS3 and NOS3 dimers were expressed as a ratio with a NOS3 monomer.

### 2.6. Statistical Analysis

Data were presented as mean ± SEM of *n* different rats. For the comparisons involving two groups, animal's significances were defined using the Mann-Whitney test. For vascular studies, a two-way ANOVA for repeated measures was used with the Bonferroni posttest. For electronic paramagnetic resonance studies, the Kruskal-Wallis test was used followed by an uncorrected Dunn's test. A value of *p* < 0.05 was considered significant. All statistical calculations and graphs were performed using GraphPad Prism software (version 8.00).

## 3. Results

### 3.1. *β*_3_-AR Overexpression in the Long Term Induces Diastolic Dysfunction

**Figure 1:**
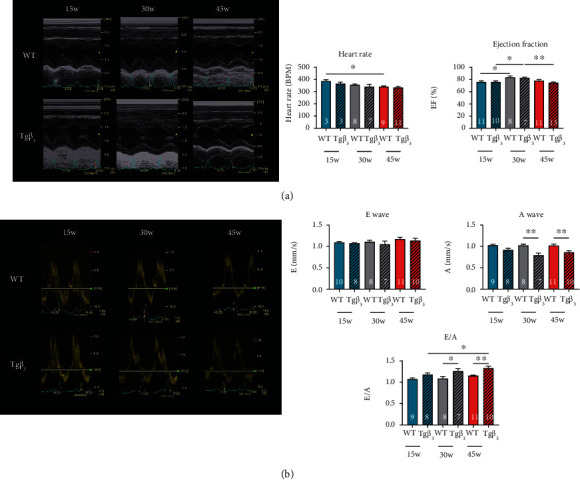
Evaluation of cardiac function in WT and Tg*β*_3_ rats at 15, 30, and 45 weeks of age. Evolution of systolic function (a) and diastolic function (b) in WT and Tg*β*_3_ rats at 15, 30, and 45 weeks of age. Values represent mean ± SEM. *n* = 3-13. ^∗^*p* < 0.05.

HFpEF is characterized by a diastolic dysfunction without major alteration of ejection fraction. In order to validate that our rat develops a diastolic dysfunction as described in Dhot et al. [10], we performed echocardiographic analyses. As shown in Figure 1, heart rate and ejection fraction were similar in both WT and Tg*β*_3_ rats at the same age (Figure 1(a)). The E wave was not changed between WT and Tg*β*_3_ rats (Figure 1(b)). The A wave was decreased in the Tg*β*_3_ group compared to the WT group at 30 and 45 weeks of age (30 weeks of age: WT: 1.02 ± 0.03; Tg*β*_3_: 0.79 ± 0.05; 45 weeks of age: WT: 1.02 ± 0.03; Tg*β*_3_: 0.86 ± 0.04; *p* < 0.05) leading to a significant increase in the E/A ratio in the Tg*β*_3_ group (30 weeks of age: WT: 1.09 ± 0.04; Tg*β*_3_: 1.26 ± 0.06; 45 weeks of age: WT: 1.15 ± 0.01; Tg*β*_3_: 1.33 ± 0.04; *p* < 0.05) (Figure 1(b)). These data validated that our model develops a diastolic dysfunction throughout ageing.

### 3.2. *β*_3_-AR Overexpression in the Long Term Induces Vasodilation Alteration

**Figure 2:**
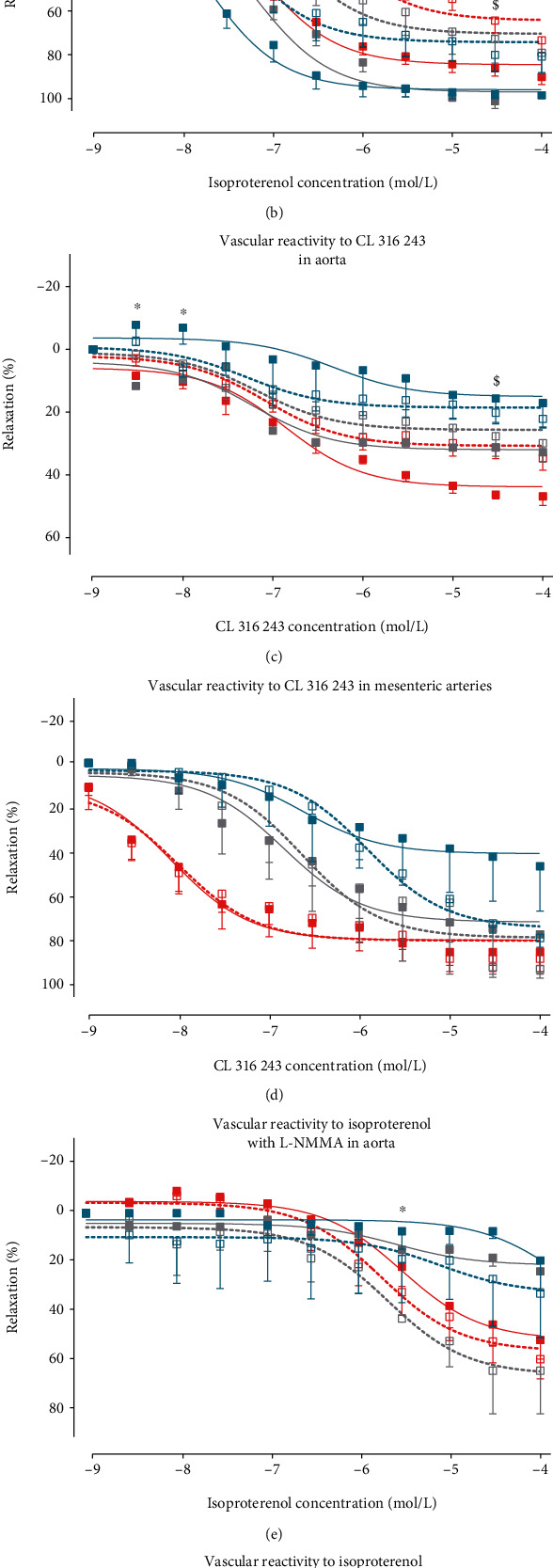
Vasodilation in WT and Tg*β*_3_ rats at 15, 30, and 45 weeks of age. Concentration-relaxation curves to isoproterenol, a nonselective *β*-AR agonist, on thoracic aortic rings (a) and mesenteric artery rings (b). Concentration-relaxation curves to CL 316 243, a *β*_3_-AR agonist, on thoracic aortic rings (c) and mesenteric artery rings (d). Concentration-relaxation curves to isoproterenol, a nonselective *β*-AR agonist, in the presence of L-NMMA, a nonselective NOS inhibitor, on thoracic aortic rings (e) and mesenteric arteries rings (f). Results were expressed as the percentage of relaxation from the maximal contraction induced by phenylephrine. Values represent mean ± SEM. *n* = 2-12. ^§^*p* < 0.05: WT—15 weeks versus Tg*β*_3_—15 weeks; ^∗^*p* < 0.05: WT—30 weeks versus Tg*β*_3_—30 weeks; ^$^*p* < 0.05: WT—45 weeks versus Tg*β*_3_—45 weeks.

Concentration-dependent vasodilation to isoproterenol, a nonselective *β*-AR agonist, was significantly reduced in aortic rings from Tg*β*_3_ rats at the age of 15 weeks (Emax; WT: 94.5 ± 2.5; Tg*β*_3_: 74.1 ± 7.5; *p* < 0.05), 30 weeks (pD_2_; WT: 6.73 ± 0.30; Tg*β*_3_: 5.49 ± 0.11; *p* < 0.05), and 45 weeks (pD_2_; WT: 6.56 ± 0.21; Tg*β*_3_: 5.82 ± 0.15; *p* < 0.05) (Figure 2(a) and Table 2(a)). In mesenteric rings, concentration-dependent vasodilation to isoproterenol was significantly reduced in Tg*β*_3_ rats at 30 weeks of age (Emax; WT: 100.7 ± 4.6; Tg*β*_3_: 73.5 ± 10.4; *p* < 0.05) and at 45 weeks of age (pD_2_; WT: 7.21 ± 0.15; Tg*β*_3_: 6.17 ± 0.17; *p* < 0.05) (Figure 2(b) and Table 2(b)). *β*3-AR overexpression seems to alter vasodilation of aortas and mesenteric arteries through the *β*-AR signaling pathway over ageing.

CL 316 243, a *β*_3_-AR agonist, produced a concentration-dependent vasodilation in both WT and Tg*β*_3_ vessels. This vasodilation was similar between the two groups of rats at all evaluated ages in both aortic and mesenteric arteries (Figures 2(c) and 2(d) and Tables 2(a) and 2(b)). Interestingly, the difference in vasodilation in response to isoproterenol between the WT and Tg*β*_3_ groups did not seem to be associated with the *β*3-AR despite its overexpression in Tg*β*_3_ rats.

To evaluate the potential involvement of the ^•^NO production through NO synthase (NOS) activity on the observed vascular dysfunction in Tg*β*_3_ rats, vasodilation induced by isoproterenol has been evaluated in the presence of L-NMMA, a nonselective NOS inhibitor. In these conditions, the vasodilation was similar between WT and Tg*β*_3_ at 15, 30, and 45 weeks of age on both thoracic aortic rings (Figure 2(e) and Table 2(a)) and mesenteric artery rings (Figure 2(f) and Table 2(b)). These results indicate that the altered vasodilation observed in Tg*β*_3_ rats at 30 and 45 weeks seems to be due to NOS activity and ^•^NO production. In particular, L-NMMA blunted the vascular relaxation in mesenteric arteries in both WT and Tg*β*_3_ rats at 45 weeks, suggesting a central role of ^•^NO in relaxation in resistance arteries at this age. Subsequently, we were interested in the involvement of different NOS isoforms in the vasodilation in order to highlight the link between *β*3-AR overexpression and ^•^NO signaling.

### 3.3. At 15 Weeks of Age, Endothelial Function Is Not Altered by Endothelial *β*_3_-AR Overexpression

As mentioned before, in our Tg*β*_3_ model, the diastolic dysfunction appeared at 30 weeks [10]. We investigated whether rats developed endothelial dysfunction over the time through the NOS expression and activity evaluation.

#### 3.3.1. The Vasodilation Is Predominantly Mediated by NOS1 in Tg*β*_3_ Rats at 15 Weeks

At 15 weeks of age, vasodilation in response to isoproterenol was only altered in aortic rings from WT and Tg*β*_3_ rats with a decrease in maximal effect in the aorta of the Tg*β*_3_ group and a significant reduced response to isoproterenol stimulation (Figure 2(a) and Table 2(a)). To decipher the implication of each NOS isoform, several NOS inhibitors: L-NIO, L-VNIO, and 1400W which inhibit NOS3, NOS1, and NOS2, respectively, have been used.

**Figure 3:**
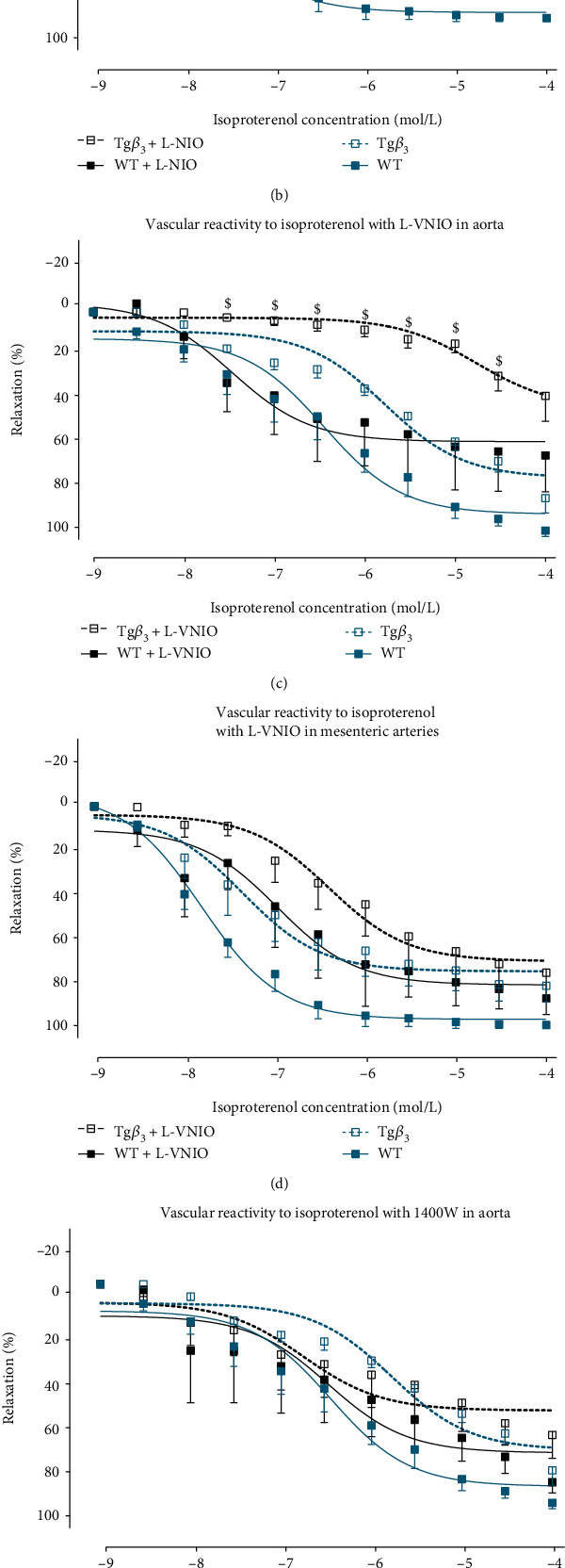
Evaluation of function of NOS in WT and Tg*β*_3_ rats at 15 weeks of age. Concentration-relaxation curves to isoproterenol, a nonselective *β*-AR agonist, on thoracic aortic rings in the presence or absence of a selective NOS inhibitor: L-NIO (NOS3 inhibitor) (a), 1400W (NOS2 inhibitor) (b), and L-VNIO (NOS1 inhibitor) (c). The same experiments were done on mesenteric artery rings with L-NIO (d), 1400W (e), and L-VNIO (f). Results were expressed as the percentage of relaxation from the maximal contraction induced by phenylephrine. Values represent mean ± SEM. *n* = 3-9. ^∗^*p* < 0.05: WT versus WT+inhibitor; ^$^*p* < 0.05: Tg*β*_3_ versus Tg*β*_3_+inhibitor.

In the presence of L-NIO, the maximal effect was reduced in the aortic rings from WT rats (WT: 60.0 ± 12.1; WT+L-NIO: 52.6 ± 7.80; *p* < 0.05) (Figure 3(a) and Table 3(a)). NOS3 inhibition using L-NIO had no significant impact on aorta and mesenteric artery reactivity from Tg*β*_3_ rats, suggesting reduced implication of ^•^NO production from NOS3 in vascular relaxation of 15-week-old Tg*β*_3_ rats compared to WT (Figures 3(a) and 3(b) and Tables 3(a) and 3(b)). In contrast, concentration-dependent vasodilation to isoproterenol in the presence of L-VNIO, a NOS1 inhibitor, was significantly decreased in the aortic rings of Tg*β*_3_ rats (Figure 3(c)), while the difference was not significant on mesenteric arteries (Figure 3(d)). In the presence of 1400W, a NOS2 inhibitor, the vasodilation in response to isoproterenol was not modified on both aortic and mesenteric rings of the two groups of rats (Figures 3(e) and 3(f) and Tables 3(a) and 3(b)). Taken together, these results suggest that vasodilation was predominantly mediated by ^•^NO produced by NOS1 in the aorta from Tg*β*_3_ rats at 15 weeks of age.

#### 3.3.2. NOS2 Expression Is Increased in Tg*β*_3_ Rats at 15 Weeks

**Figure 4:**
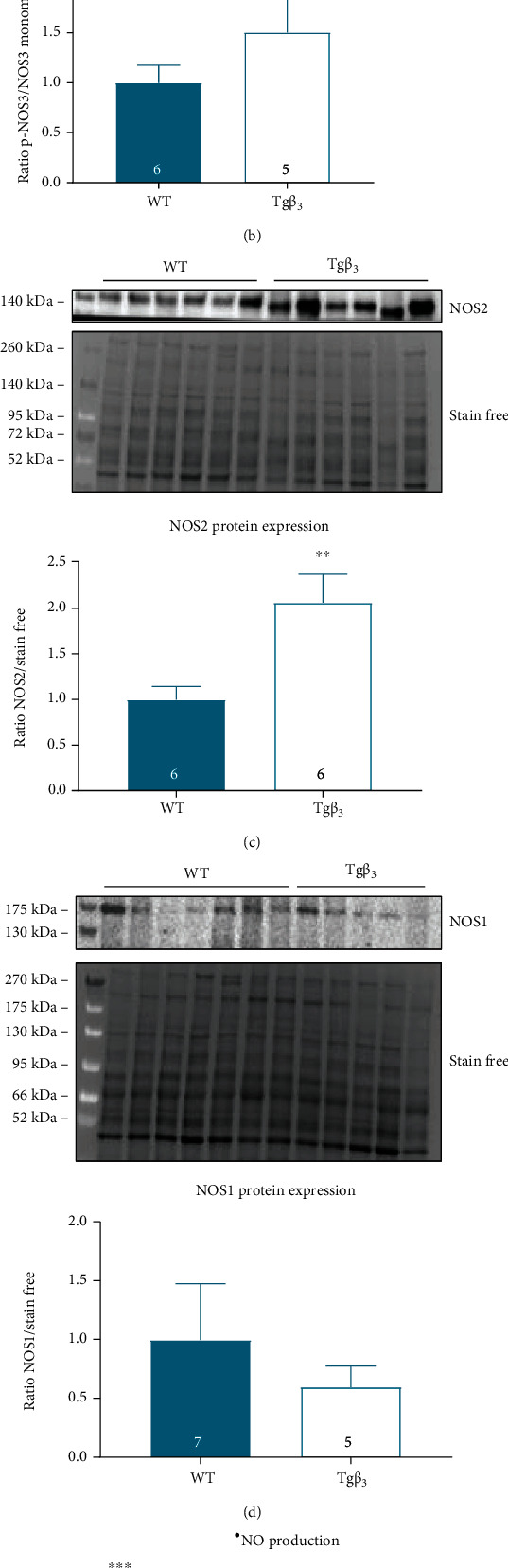
Expression and activity production of NOS in WT and Tg*β*_3_ rats at 15 weeks of age. Effects of the *β*_3_-AR overexpression in Tg*β*_3_ rats on aortic protein expression of the NOS3 dimer/monomer ratio (a), p-NOS3/NOS3 monomer (b), NOS2 (c), and NOS1 (d). Evaluation of ^•^NO production on thoracic aortic rings with or without an inhibitor (L-NMMA, L-NIO, 1400W, and L-VNIO) (e) and O_2_^•-^ production with or without L-NMMA from WT and Tg*β*_3_ rats (f). Data are expressed as mean ± SEM. *n* = 5-14. ^∗∗^*p* < 0.01, ^∗∗∗^*p* < 0.001.

The aortic ratio of NOS3 dimer/monomer and p-NOS3/NOS3 monomer protein expression levels and NOS1 expression was not significantly modified at 15 weeks of age (Figures 4(a), 4(b), and 4(d)). The overexpression of *β*_3_-AR induced a significant 2-fold increase in NOS2 protein expression in the aorta (WT: 1.00 ± 0.14; Tg*β*_3_: 2.06 ± 0.31; *p* < 0.05) (Figure 4(c)).

#### 3.3.3. ^•^NO and O_2_^•-^ Production Is Unchanged in the Tg*β*_3_ Rats at 15 Weeks

^•^NO production remained stable on thoracic aortas between WT and transgenic rats. With L-NMMA, the ^•^NO production was almost abolished as expected, confirming that NOS-dependent ^•^NO production (negative control). The NOS2-dependent ^•^NO production was not significantly increased in the Tg*β*_3_ rats (+51%; *p* = 0.09). The NOS1- and NOS3-dependent ^•^NO production was not modified between the two groups (Figure 4(e)). The generation of O_2_^•-^ has been also evaluated under baseline conditions and in the presence of L-NMMA, and no changes on O_2_^•-^ production with or without L-NMMA were reported between the two groups of rats at this age (Figure 4(f)).

### 3.4. *β*_3_-AR Overexpression Induced Endothelial Dysfunction at 30 Weeks

We investigated whether endothelial dysfunction is worsened at 30 weeks of age in Tg*β*_3_ rats.

#### 3.4.1. NOS1 Modulates the Vasodilatation in the Tg*β*_3_ Group

At 30 weeks of age, vasodilation in response to isoproterenol was reduced in aortic rings of Tg*β*_3_ rats associated with a decreased pD_2_ (WT: 6.73 ± 0.30; Tg*β*_3_: 5.49 ± 0.11; *p* < 0.05) (Figure 2(a) and Table 2(a)).

**Figure 5:**
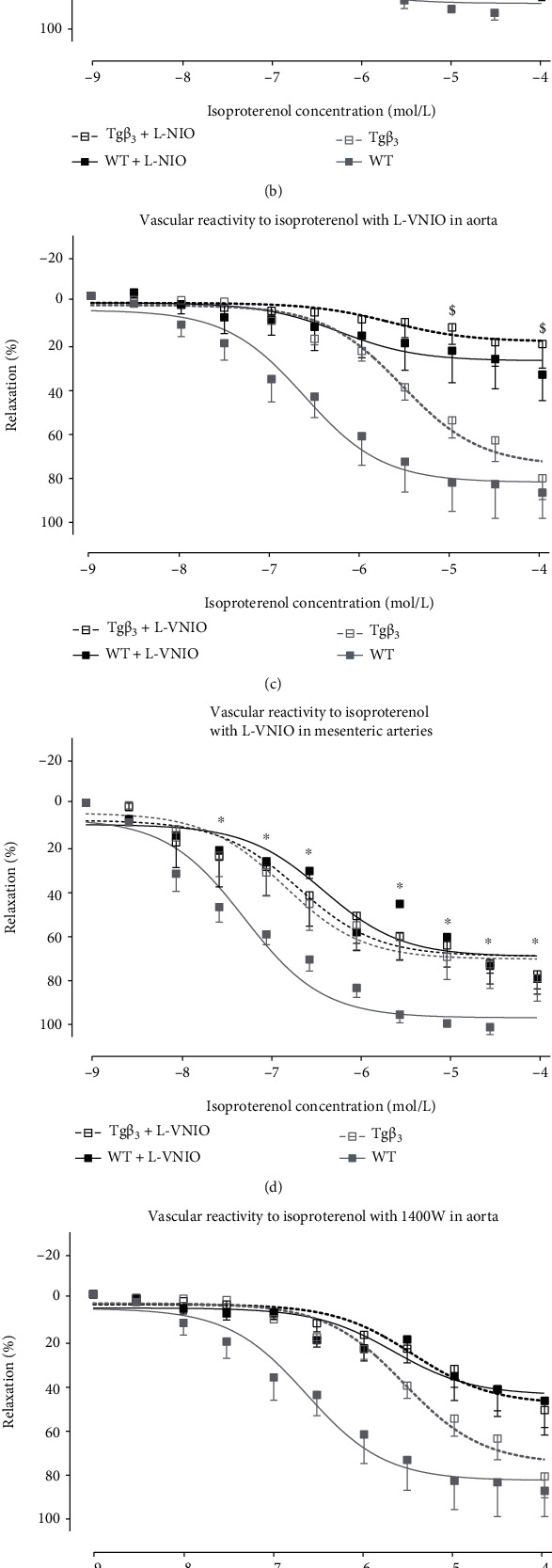
Evaluation of function of NOS in WT and Tg*β*_3_ rats at 30 weeks of age. Concentration-relaxation curves to isoproterenol, a nonselective *β*-AR agonist, on thoracic aortic rings in the presence or absence of a selective NOS inhibitor: L-NIO (NOS3 inhibitor) (a), 1400W (NOS2 inhibitor) (b), and L-VNIO (NOS1 inhibitor) (c). The same experiments were done on mesenteric artery rings with L-NIO (d), 1400W (e), and L-VNIO (f). Results were expressed as the percentage of relaxation from the maximal contraction induced by phenylephrine. Values represent mean ± SEM. *n* = 3-8. ^∗^*p* < 0.05: WT versus WT+inhibitor; ^$^*p* < 0.05: Tg*β*_3_ versus Tg*β*_3_+inhibitor.

In the presence of L-NIO, the pD_2_ was reduced in the aortic rings (WT: 6.73 ± 0.30; WT+L-NIO: 5.25 ± 0.14; *p* < 0.05) and mesenteric arteries (WT: 7.03 ± 0.20; WT+L-NIO: 5.91 ± 0.31; *p* < 0.05) from WT rats (Figures 5(a) and 5(b) and Tables 3(a) and 3(b)) suggesting the implication of ^•^NO from NOS3.

Vasodilation in response to isoproterenol in the presence of the NOS1 inhibitor (L-VNIO) was blunted with a reduction in maximal effect in the aortic rings from both WT rats (WT: 85.6 ± 14.3; WT+L-NIO: 35.25 ± 11.19; *p* < 0.05) and Tg*β*_3_ rats (Tg*β*_3_: 79 ± 9.5; Tg*β*_3_+L-NIO: 18.6 ± 8.6; *p* < 0.05) and a decrease in the maximal effect in the mesenteric arteries from WT rats (WT: 100.7 ± 4.6; WT+L-NIO: 71.5 ± 4.59; *p* < 0.05) (Figures 5(c) and 5(d) and Tables 3(a) and 3(b)). These data suggest an implication of ^•^NO production from NOS1.

In the presence of 1400W, the vasodilation in response to isoproterenol was modified on the aorta from WT rats with reduced maximal effect (WT: 85.6 ± 14.3; WT+1400W: 45.58 ± 13.7; *p* < 0.05) (Figure 5(e) and Table 3(a)) with no impact on mesenteric arteries (Figure 5(f) and Table 3(b)). At 30 weeks of age, the vasodilation induced by isoproterenol seems to involve all NOS isoforms in WT rats while only NOS1 seems to be involved in the vasodilation of vessels from Tg*β*_3_ rats.

#### 3.4.2. *β*_3_-AR Overexpression Is Associated with an Increase in NOS1 Expression

**Figure 6:**
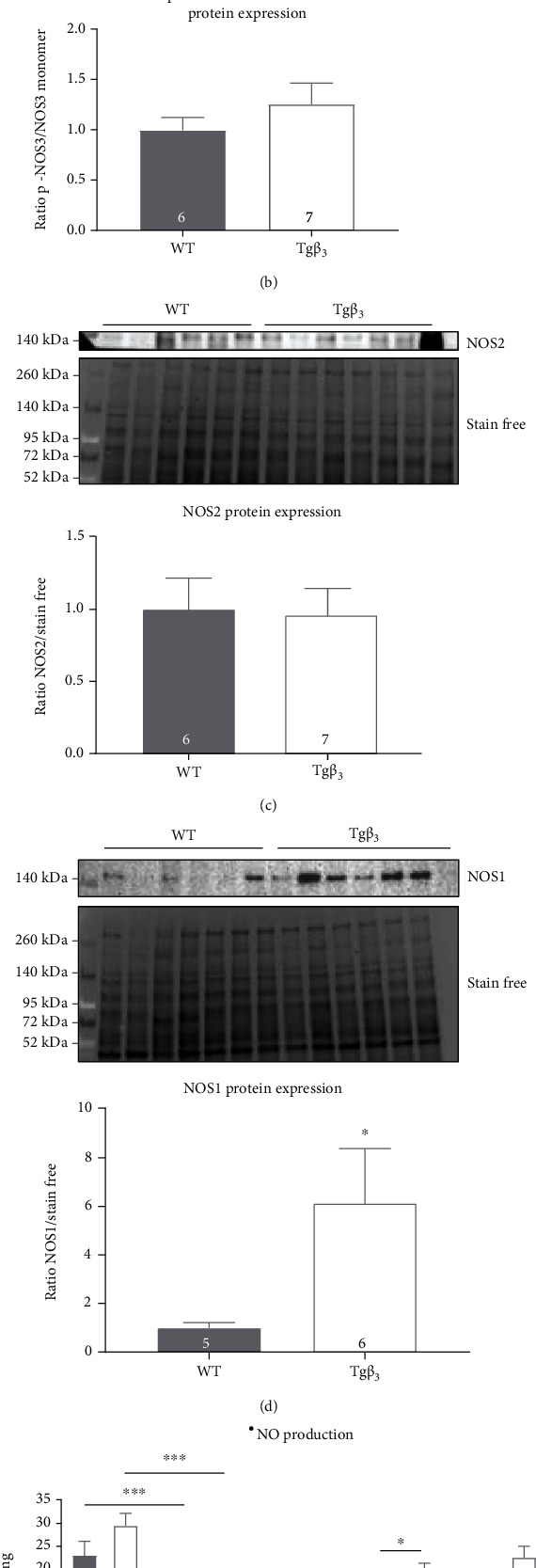
Expression and activity production of NOS in WT and Tg*β*_3_ rats at 30 weeks of age. Effects of the *β*_3_-AR overexpression in Tg*β*_3_ rats on aortic protein expression of the NOS3 dimer/monomer ratio (a), p-NOS3/NOS3 monomer (b), NOS2 (c), and NOS1 (d). Evaluation of ^•^NO production on thoracic aortic rings with or without an inhibitor (L-NMMA, L-NIO, L-VNIO, and 1400W) (e) and O_2_^•-^ production with or without L-NMMA (f) from WT and Tg*β*_3_ rats. Data are expressed as mean ± SEM. *n* = 5-16. ^∗^*p* < 0.05, ^∗∗∗^*p* < 0.001.

The aortic ratio of the NOS3 dimer/monomer and p-NOS3/NOS3 monomer and NOS2 protein expression levels were not significantly modified between the two groups at 30 weeks (Figures 6(a), 6(b), and 6(c)). The *β*_3_-AR overexpression induces a significant 6-fold increase in NOS1 protein expression in the aorta (Figure 6(d)) which can explain the decrease in vasodilation in vessels from Tg*β*_3_ with the NOS1 inhibitor.

#### 3.4.3. Oxidative Stress Is Increased in Tg*β*3 Rats at 30 Weeks

At 30 weeks of age, ^•^NO production tends to increase in Tg*β*_3_ rats (+28%). In the presence of L-NMMA, the production of ^•^NO is significantly reduced in WT and Tg*β*_3_ rats. NOS2-dependent ^•^NO production is increased in the Tg*β*_3_ group (+70% vs. WT; *p* < 0.05) (Figure 6(e)).

The O_2_^•-^ production was significantly increased in the Tg*β*_3_ group under baseline conditions (+75% vs. WT; *p* < 0.05) while no change is shown with L-NMMA (Figure 6(f)).

The results demonstrated that despite a similar expression of NOS2, NOS2-dependent ^•^NO production is increased in the Tg*β*_3_ group, suggesting that its activity is enhanced in Tg*β*_3_ rats. Interestingly, NOS1 expression which is highly increased in Tg*β*_3_ rats compared to WT does not impact ^•^NO production.

### 3.5. *β*_3_-AR Overexpression Maintained the Endothelial Dysfunction at 45 Weeks of Age

At 30 weeks, results indicated an endothelial dysfunction associated with an alteration of the vasodilation. An increase in NOS1 expression could led to endothelial dysfunction and is associated with an increase in O_2_^•-^ production. Thus, we investigated, at 45 weeks of age, whether endothelial dysfunction was more severe compared to 30 weeks of age.

#### 3.5.1. NOS1 and NOS3 Modulate the Vasodilation in the Tg*β*_3_ Group

**Figure 7:**
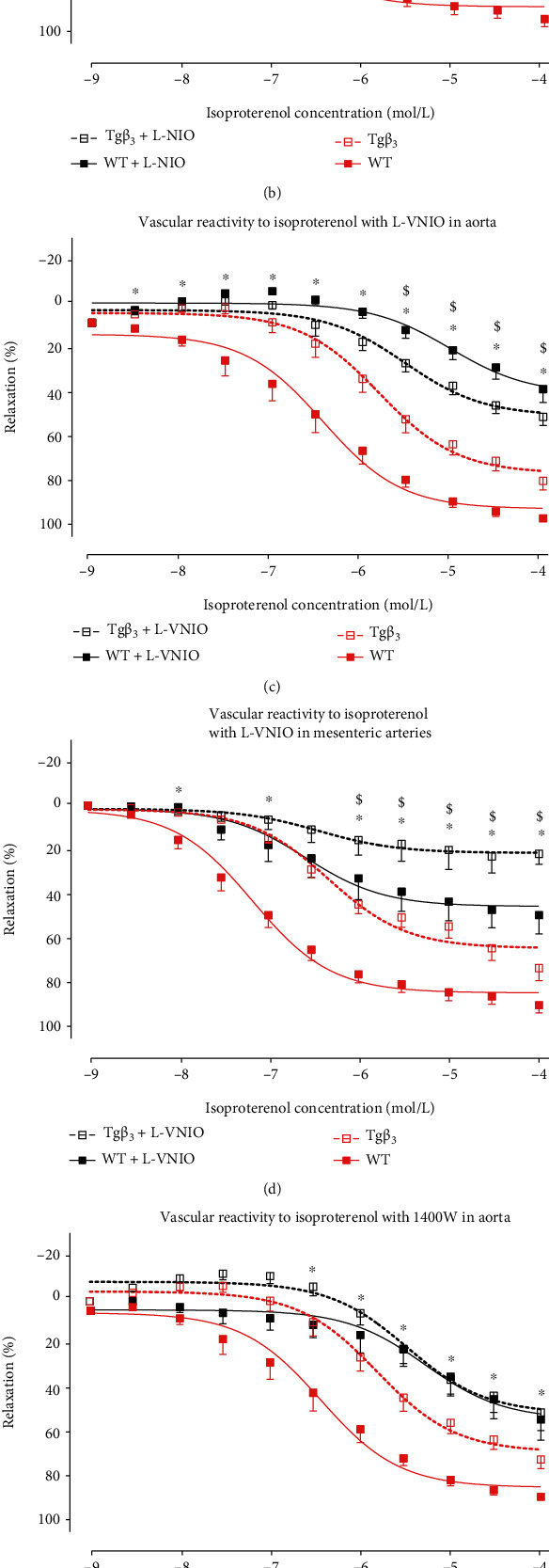
Evaluation of function of NOS in WT and Tg*β*_3_ rats at 45 weeks of age. Concentration-relaxation curves to isoproterenol, a nonselective *β*-AR agonist, on thoracic aortic rings in the presence or absence of a selective NOS inhibitor: L-NIO (NOS3 inhibitor) (a), 1400W (NOS2 inhibitor) (b), and L-VNIO (NOS1 inhibitor) (c). The same experiments were done on mesenteric artery rings with L-NIO (d), 1400W (e), and L-VNIO (f). Results were expressed as the percentage of relaxation from the maximal contraction induced by phenylephrine. Values represent mean ± SEM. *n* = 5-12. ^∗^*p* < 0.05: WT versus WT+inhibitor; ^$^*p* < 0.05: Tg*β*_3_ versus Tg*β*_3_+inhibitor.

At 45 weeks of age, vasodilation in response to isoproterenol was significantly reduced in Tg*β*_3_ rats compared to WT in both aortic and mesenteric arteries (Figures 2(a) and 2(b) and Tables 2(a) and 2(b)). In the presence of L-NIO, 1400W, or L-VNIO, vasodilation was significantly reduced on aortic and mesenteric artery rings from WT rats (Figures 7(a)–7(f) and Tables 3(a) and 3(b)). In Tg*β*_3_ rats, vasodilation was significantly reduced on aortic rings in the presence of L-NIO, while L-VNIO reduced the vasodilation in both aortic and mesenteric rings from Tg*β*_3_ rats (Figures 7(a), 7(c), and 7(d)). Taken together, these results demonstrated that the vasodilation in Tg*β*_3_ rats was altered compared to that in WT rats and seems to depend on NOS1 and NOS3 isoforms.

#### 3.5.2. NOS3 Is Uncoupled in Tg*β*_3_ Rats

**Figure 8:**
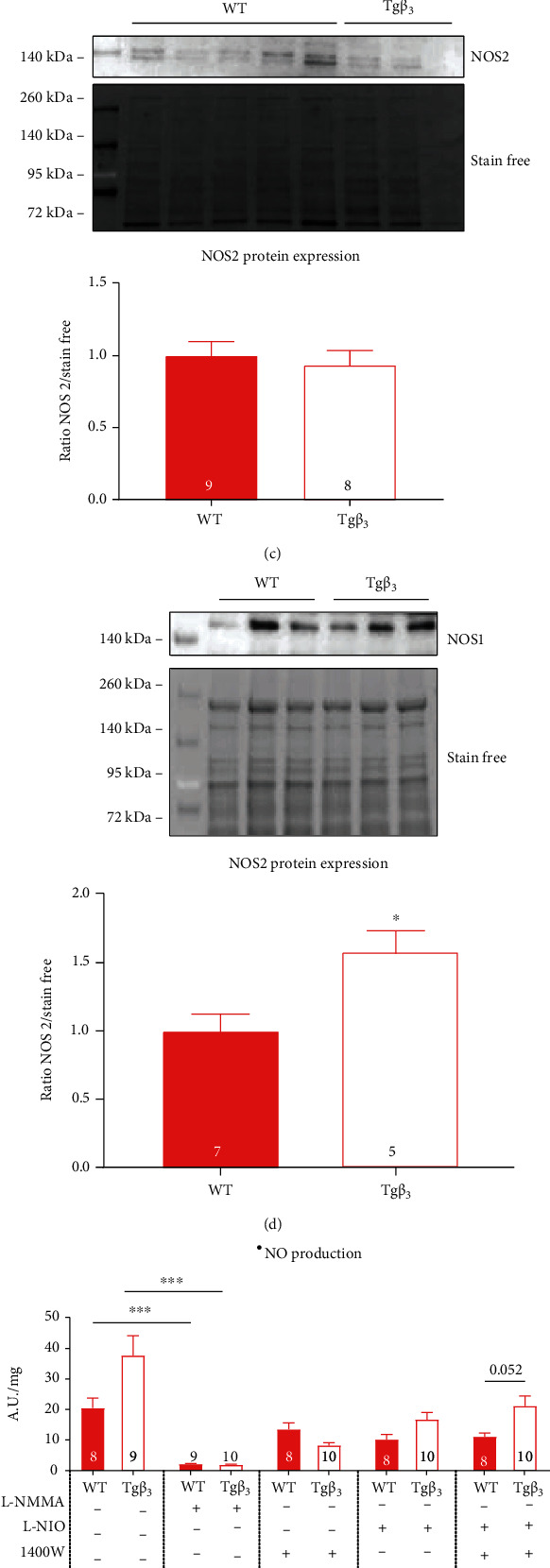
Expression and activity production of NOS in WT and Tg*β*_3_ rats at 45 weeks of age. Effects of the *β*_3_-AR overexpression in Tg*β*_3_ rats on aortic protein expression of the NOS3 dimer/monomer ratio (a), p-NOS3/NOS3 monomer (b), NOS2 (c), and NOS1 (d). Evaluation of ^•^NO production on thoracic aortic rings with or without an inhibitor (L-NMMA, L-NIO, L-VNIO, and 1400W) (e) and O_2_^•-^ production with or without L-NMMA (f) from WT and Tg*β*_3_ rats. Data are expressed as mean ± SEM. *n* = 5-10. ^∗^*p* < 0.05, ^∗∗^*p* < 0.01, and ^∗∗∗^*p* < 0.001.

In Tg*β*_3_ rats, the aortic NOS3 dimer/NOS3 monomer protein expression ratio and the aortic p-NOS3/NOS3 monomer protein expression ratio were significantly reduced (-87% and -63%, respectively; *p* < 0.05) indicating a potential NOS3 uncoupling (Figures 8(a) and 8(b)). No modification was reported for NOS2 protein expression (Figure 8(c)). The aortic NOS1 protein expression was significantly increased at 45 weeks of age on Tg*β*_3_ rats (+57%; *p* < 0.05) (Figure 8(d)).

#### 3.5.3. O_2_^•-^ Production Is Increased in Tg*β*_3_ Rats

The production of ^•^NO tends to increase in the aorta in Tg*β*_3_ rats under baseline conditions (+84% vs. WT) (Figure 8(e)). Addition of L-NMMA reduces ^•^NO production in the two groups, indicating that the ^•^NO production from Tg*β*_3_ and WT rats is mainly due to the NOS. The NOS1-dependent ^•^NO production tends to increase in the Tg*β*_3_ rats (+90% vs. WT; *p* = 0.052) (Figure 8(e)). The production of O_2_^•-^ was significantly increased in Tg*β*_3_ rats (+76% vs. WT; *p* < 0.05). L-NMMA treatment normalized the production with the absence of significant difference between the two groups, suggesting the involvement of NOS in O_2_^•-^ production from Tg*β*_3_ rats (Figure 8(f)). Results obtained at 45 weeks of age confirm the previous results obtained at 30 weeks of age that Tg*β*_3_ rats present an endothelial dysfunction.

## 4. Discussion

The aim of this study was to decipher the link between endothelial dysfunction and diastolic dysfunction. We wanted to understand if endothelial dysfunction could be involved in HFpEF development. For that, we investigated cardiac and endothelial function in rats at 15, 30, and 45 weeks old of age. The major finding of this study was that *β*_3_-AR overexpression led to endothelial dysfunction throughout ageing and that endothelium dysfunction appears prior to diastolic dysfunction.

### 4.1. *β*_3_-AR Overexpression Is Associated with Endothelial Dysfunction

*Adrb3*, the gene coding for the *β*3 receptor, is the main subtype of the *β* adrenergic receptor expressed in the whole aorta in the rats. However, smooth muscle cells and endothelial cells are mainly expressing the gene coding for the *β*2 receptor (*Adrb2*) [16]. In the literature, *β*_3_-AR has a low level of protein expression under physiological conditions, but *β*_3_-AR expression is increased in heart failure [17], and many studies demonstrated a cardioprotective role of *β*_3_-AR in pathophysiological conditions [18–22]. Also, specific *β*_3_-AR overexpression on cardiomyocytes is associated with the cardioprotective effect [23]. In our study however, long-term endothelial overexpression of the *β*_3_-AR was associated with deleterious effect such as the development of diastolic dysfunction and an alteration of the vascular function. Vascular reactivity, and specifically vasodilation in response to isoproterenol, was blunted as early as 15 weeks of age, before the apparition of the diastolic dysfunction, suggesting that endothelial dysfunction is a key process in the HFpEF development. Interestingly, altered vasodilation was first detected in the aorta at 15 weeks while it has been detected in both the aorta and mesenteric arteries at 30 weeks. This can be explained in particular by structural differences but also by differences in ^•^NO production between elastic arteries such as the aorta and muscular arteries such as the mesenteric arteries [24].

### 4.2. Endothelial Dysfunction and HFpEF

Endothelial dysfunction has been suggested to be at the center of the HFpEF pathophysiology for about ten years [25–27], yet no study manages to demonstrate that endothelial dysfunction could be the *primum movens* of HFpEF development.

Endothelial dysfunction has been characterized in HFpEF by a decrease in the production of ^•^NO, leading to a decrease in vasodilation, as a consequence of the NOS3 protein loss of function [25]. The decrease in ^•^NO is explained by the decoupling of NOS3, which has been confirmed in patients with HFpEF or in animal models such as ZSF1 (Zucker fatty/spontaneously hypertensive heart failure F1) rats. NOS3 uncoupling leads to a shift of NOS3 from the dimer, which produces ^•^NO, to the monomer, which generates O_2_^•-^ [28]. This NOS3 uncoupling is also found in a diabetic HFpEF model [29]. In their study, the authors show a decrease in the activity of NOS3 due to uncoupling of this protein and thus a decrease in the production of ^•^NO. Interestingly, Shibata et al. have shown that, by using NOS-knockout mice, the deletion of NOS causes diastolic dysfunction and cardiac hypertrophy [30]. These studies converge to demonstrate that during HFpEF development, the uncoupling of the NOS3 protein induces a decrease in ^•^NO production, which could be at the origin of endothelial dysfunction.

NOS1 is largely expressed in the central nervous system, but its expression has also been found in smooth muscle cells and cardiomyocytes. Under physiological conditions, NOS1 expression in smooth muscle cells has been shown to be involved in vascular tone. Cau et al. described that NOS-dependent vasodilation is modified throughout ageing in human and animal models. In fact, NOS3 expression and activity are reduced with age, whereas NOS2 activity is increased and accompanied by peroxynitrite production [31]. These data validate our observations on the involvement of NOS isoforms in vasodilation throughout ageing, with a predominant involvement of NOS3 at 15 weeks of age and all NOS isoforms at 30 and 45 weeks of age. Interestingly, NOS1 has been shown to maintain some degree of vasodilation, when the predominant NOS3 becomes dysfunctional [32, 33] or absent in the knockout mouse model [34, 35]. In our Tg*β*_3_ rat model, we showed that NOS1 expression was increased at 30 and 45 weeks of age and that the NOS1 is the main isoform involved in the vasodilation. These data suggest that NOS1 could compensate for the dysfunction of NOS3 reported at 45 weeks of age.

### 4.3. Oxidative Stress: A Key Player in Endothelial and Diastolic Dysfunction

Oxidative stress in another mechanism recently described a potential trigger to the development of endothelial dysfunction. Indeed, it has been described that the H_2_O_2_ concentration in the myocardium of both HFpEF patients and ZSF1 rats is significantly elevated [25]. H_2_O_2_ results from the conversion of O_2_^•-^ by superoxide dismutase (SOD), and the high O_2_^•-^ concentrations in our model therefore suggest an increase in H_2_O_2_ concentration and SOD activity at the vascular and cardiac levels.

The endothelial dysfunction is linked to an increase in ^•^NO production by NOS1. In the same time, NOS3 expression is decreased suggesting NOS3 uncoupling leading to O_2_^•-^ production and oxidative stress as described earlier [9, 36]. Increase in oxidative stress is linked to the genesis of heart failure, but some studies suggested that *β*_3_-AR activation inhibited oxidative stress and reactive oxygen production (ROS). Intriguingly, in our model, long-term endothelial *β*_3_-AR overexpression did not protect against the ROS production, and the long-term overproduction of ^•^NO is linked to endothelial dysfunction. Several hypotheses can explain this phenomenon. First, the increase in S-nitrosylation could explain the progressive decrease in cardiovascular function. Recently, Schiattarella et al. showed that elevated NOS2 activity leads to S-nitrosylation in multiple proteins and can impair their functions. Furthermore, the study highlighted, in human HFpEF hearts, an increase in NOS2 transcripts [27]. In our study, NOS2 expression was increased as early as 15 weeks of age in the Tg*β*_3_ group suggesting a disruption of protein function. However, nothing can be concluded since the expression of a protein and the enzymatic activity of the latter are not always linked. In the second hypothesis, the increase in the ^•^NO bioavailability associated with an increase in ROS could lead to the production of peroxynitrite (ONOO^−^). In our study, the trend to increase in ^•^NO levels is associated with the overproduction of O_2_^•-^ suggesting the production of ONOO^−^. The production of ONOO^−^ is increased when the NOS3 is uncoupled [37]. In our model, the decrease in O_2_^•-^ production in the presence of L-NMMA suggested that the NOS uncoupling could be at the origin of a reduced vasodilation. More specifically, the uncoupled NOS3, reported in the Tg*β*_3_ rats, could be at the origin of the diastolic dysfunction. Oxidative stress and more particularly NOS impairment could have a key role in endothelial dysfunction and with ageing could lead to cardiac alteration.

## 5. Limits

The aim of the study is to better understand the role of endothelial dysfunction in the development of HFpEF. HFpEF is a disease that primarily affects women. In our model of human *β*3-adrenoreceptor overexpression and as discussed in our previous publication [10], only male rats develop heart failure with preserved ejection fraction. From our results, the involvement of the endothelium in the development of HFpEF could only be linked to the male phenotype.

## 6. Conclusion

Endothelial dysfunction appeared prior to cardiac dysfunction in our HFpEF rat model indicating a potential role of endothelial dysfunction in the development of HFpEF. We have demonstrated that alteration in the NOS function was a potential trigger of HFpEF development via an endothelial dysfunction. The increase in oxidative stress was characterized by an increase in O_2_^•-^ which was the consequence of NOS deregulation in our model potentially via NOS3 uncoupling. Indeed, in many HFpEF models, the NOS3 protein is described as uncoupled, resulting in a decrease in the production of ^•^NO and an increase in O_2_^•-^, yet as a consequence of HFpEF and not as a cause. Our study provides evidence that endothelial dysfunction could be the trigger to develop HFpEF.

## Figures and Tables

**Table 1:** Antibodies used for Western blotting experiments.

Protein of interest	Primary antibody			Secondary antibody		
	Reference	Species	Dilution	Reference	Species	Dilution
NOS1	Cell Signaling (4231)	Rabbit	1/500^∗^	Cell Signaling (7074)	Goat	1/10000^∗^
NOS2	Millipore (AB5382)	Rabbit	1/2000^∗^			
NOS3	BD Biosciences (610296)	Mouse	1/1000^∗^	Cell Signaling (7076)	Horse	
p-NOS3	Cell Signaling (9571)	Rabbit	1/1000^∗^	Cell Signaling (7074)	Goat	

^∗^Dilution carried out in 5% milk.

**(a):** 

Group		Thoracic aortic arteries—WT			Thoracic aortic arteries—Tg*β*_3_			Statistics WT vs. Tg*β*_3_	
		*n*	pD_2_	Emax	*n*	pD_2_	Emax	p pD_2_	p Emax
Isoproterenol	15 w	7	6.53 ± 0.33	94.5 ± 2.5	5	5.93 ± 0.20	74.1 ± 7.5	0.239	0.015
	30 w	4	6.73 ± 0.30	85.6 ± 14.3	7	5.49 ± 0.11	79.0 ± 9.5	0.002	0.245
	45 w	11	6.56 ± 0.21	85.9 ± 2.2	10	5.82 ± 0.15	69.2 ± 3.5	0.023	0.016
Isoproterenol+L-NMMA	15 w	3	5.18 ± 0.80	15.0 ± 5.52	2	6.79 ± 2.07	25.7 ± 9.43	0.408	0.679
	30 w	2	5.62 ± 0.11	19.9 ± 5.40	3	5.79 ± 0.34	63.4 ± 18.5	0.981	0.080
	45 w	9	5.46 ± 0.24	51.9 ± 7.27	8	5.62 ± 0.22	56.0 ± 6.55	0.697	0.760
Isoproterenol+L-NIO	15 w	7	6.06 ± 0.45	60.0 ± 12.1	4	5.87 ± 0.43	52.6 ± 7.80	0.756	0.491
	30 w	3	5.25 ± 0.14	57.0 ± 22.8	6	5.27 ± 0.27	42.1 ± 14.3	0.537	0.647
	45 w	8	4.93 ± 0.11	33.0 ± 4.42	8	5.30 ± 0.18	45.9 ± 5.90	0.212	0.278
Isoproterenol+L-VNIO	15 w	4	6.79 ± 0.638	61.9 ± 16.0	5	5.63 ± 0.512	47.9 ± 21.6	0.118	0.449
	30 w	4	5.65 ± 0.63	35.25 ± 11.9	4	6.02 ± 0.59	18.6 ± 8.6	0.572	0.530
	45 w	5	4.97 ± 0.14	33.6 ± 6.15	5	5.54 ± 0.15	42.3 ± 2.84	0.186	0.702
Isoproterenol+1400W	15 w	4	6.51 ± 0.700	83.2 ± 7.23	4	6.32 ± 0.656	65.7 ± 8.77	0.905	0.151
	30 w	4	6.09 ± 0.41	45.58 ± 13.7	7	5.26 ± 0.32	63.1 ± 12.6	0.213	0.296
	45 w	7	5.43 ± 0.46	58.1 ± 9.44	8	5.35 ± 0.13	53.0 ± 7.52	0.896	0.589
CL 316 243	15 w	3	6.16 ± 0.67	17.1 ± 6.9	4	6.75 ± 0.74	22.8 ± 2.5	0.197	0.533
	30 w	2	7.22 ± 0.00	32.0 ± 0.0	3	6.87 ± 0.45	26.3 ± 5.0	0.785	0.442
	45 w	4	6.94 ± 0.20	43.7 ± 1.9	9	7.06 ± 0.09	30.9 ± 3.8	0.646	0.044

**(b):** 

Group		Mesenteric arteries—WT			Mesenteric arteries—Tg*β*_3_			Statistics WT vs. Tg*β*_3_	
		*n*	pD_2_	Emax	*n*	pD_2_	Emax	p pD_2_	p Emax
Isoproterenol	15 w	4	7.78 ± 0.17	96.4 ± 3.0	8	7.15 ± 0.32	78.0 ± 8.5	0.112	0.151
	30 w	8	7.03 ± 0.20	100.7 ± 4.6	8	6.67 ± 0.28	73.5 ± 10.4	0.338	0.022
	45 w	12	7.21 ± 0.15	85.7 ± 3.4	12	6.17 ± 0.17	68.2 ± 5.5	0.001	0.061
Isoproterenol+L-NMMA	15 w	4	6.67 ± 0.49	83.8 ± 14.2	6	6.82 ± 0.53	87.5 ± 8.25	0.877	0.920
	30 w	7	6.37 ± 0.50	92.1 ± 4.60	6	5.72 ± 0.15	86.1 ± 7.93	0.392	0.815
	45 w	9	6.33 ± 0.47	5.07 ± 4.53	8	6.38 ± 0.47	−4.03 ± 2.60	0.918	0.533
Isoproterenol+L-NIO	15 w	3	6.04 ± 0.78	86.1 ± 1.28	9	6.89 ± 0.30	75.4 ± 8.08	0.300	0.646
	30 w	5	5.91 ± 0.31	76.9 ± 4.70	4	6.66 ± 0.38	86.8 ± 5.66	0.195	0.344
	45 w	9	5.83 ± 0.29	35.1 ± 5.91	9	5.65 ± 0.36	64.0 ± 7.98	0.818	0.033
Isoproterenol+L-VNIO	15 w	6	7.08 ± 0.52	84.1 ± 8.64	6	6.24 ± 0.45	75.8 ± 9.94	0.221	0.744
	30 w	3	6.29 ± 0.27	71.5 ± 4.59	6	6.78 ± 0.54	75.1 ± 8.33	0.491	0.564
	45 w	8	6.41 ± 0.30	49.6 ± 8.63	8	6.82 ± 0.43	22.79 ± 7.38	0.392	0.171
Isoproterenol+1400W	15 w	4	7.34 ± 0.25	96.4 ± 2.02	7	6.49 ± 0.31	81.8 ± 8.00	0.082	0.515
	30 w	8	6.64 ± 0.30	84.0 ± 10.9	6	6.43 ± 0.44	92.3 ± 3.23	0.759	0.789
	45 w	8	6.32 ± 0.40	46.52 ± 10.8	8	6.27 ± 0.29	52.7 ± 13.8	0.781	0.325
CL 316 243	15 w	5	5.98 ± 0.61	48.4 ± 19.5	6	5.90 ± 0.14	75.7 ± 5	0.654	0.856
	30 w	3	6.53 ± 0.64	75.2 ± 16.8	4	6.52 ± 0.65	93.2 ± 14.5	0.968	0.568
	45 w	3	7.96 ± 0.33	81.1 ± 7.4	7	7.68 ± 0.42	84.5 ± 5.8	0.731	0.737

**(a):** 

Group		Thoracic aortic arteries—WT			Thoracic aortic arteries—Tg*β*_3_			WT vs. WT+isoproterenol		Tg*β*_3_ vs. Tg*β*_3_+isoproterenol	
		*n*	pD_2_	Emax	*n*	pD_2_	Emax	p pD_2_	p Emax	p pD_2_	p Emax
Isoproterenol	15 w	7	6.53 ± 0.33	94.5 ± 2.5	5	5.93 ± 0.20	74.1 ± 7.5				
	30 w	4	6.73 ± 0.30	85.6 ± 14.3	7	5.49 ± 0.11	79.0 ± 9.5
	45 w	11	6.56 ± 0.21	85.9 ± 2.2	10	5.82 ± 0.15	69.2 ± 3.5
Isoproterenol+L-NIO	15 w	7	6.06 ± 0.45	60.0 ± 12.1	4	5.87 ± 0.43	52.6 ± 7.80	0.430	0.013	0.939	0.322
	30 w	3	5.25 ± 0.14	57.0 ± 22.8	6	5.27 ± 0.27	42.1 ± 14.3	0.006	0.083	0.661	0.080
	45 w	8	4.93 ± 0.11	33.0 ± 4.42	8	5.30 ± 0.18	45.9 ± 5.90	<0.0001	<0.0001	0.134	0.088
Isoproterenol+L-VNIO	15 w	4	6.79 ± 0.638	61.9 ± 16.0	5	5.63 ± 0.512	47.9 ± 21.6	0.620	0.125	0.799	0.414
	30 w	4	5.65 ± 0.63	35.25 ± 11.9	4	6.02 ± 0.59	18.6 ± 8.6	0.090	0.024	0.346	0.019
	45 w	5	4.97 ± 0.14	33.6 ± 6.15	5	5.54 ± 0.15	42.3 ± 2.84	<0.0001	<0.0001	0.377	0.068
Isoproterenol+1400W	15 w	4	6.51 ± 0.700	83.2 ± 7.23	4	6.32 ± 0.656	65.7 ± 8.77	0.665	0.259	0.588	0.623
	30 w	4	6.09 ± 0.41	45.58 ± 13.7	7	5.26 ± 0.32	63.1 ± 12.6	0.191	0.019	0.902	0.323
	45 w	7	5.43 ± 0.46	58.1 ± 9.44	8	5.35 ± 0.13	53.0 ± 7.52	0.002	0.002	0.105	0.265

**(b):** 

Group		Mesenteric arteries—WT			Mesenteric arteries—Tg*β*_3_			WT vs. WT+isoproterenol		Tg*β*_3_ vs. Tg*β*_3_+isoproterenol	
		*n*	pD_2_	Emax	*n*	pD_2_	Emax	p pD_2_	p Emax	p pD_2_	p Emax
Isoproterenol	15 w	4	7.78 ± 0.17	96.4 ± 3.0	8	7.15 ± 0.32	78.0 ± 8.5				
	30 w	8	7.03 ± 0.20	100.7 ± 4.6	8	6.67 ± 0.28	73.5 ± 10.4
	45 w	12	7.21 ± 0.15	85.7 ± 3.4	12	6.17 ± 0.17	68.2 ± 5.5
Isoproterenol+L-NIO	15 w	3	6.04 ± 0.78	86.1 ± 1.28	9	6.89 ± 0.30	75.4 ± 8.08	0.024	0.431	0.555	0.776
	30 w	5	5.91 ± 0.31	76.9 ± 4.70	4	6.66 ± 0.38	86.8 ± 5.66	0.014	0.003	0.978	0.627
	45 w	9	5.83 ± 0.29	35.1 ± 5.91	9	5.65 ± 0.36	64.0 ± 7.98	0.0004	<0.0001	0.408	0.640
Isoproterenol+L-VNIO	15 w	6	7.08 ± 0.52	84.1 ± 8.64	6	6.24 ± 0.45	75.8 ± 9.94	0.290	0.361	0.127	0.844
	30w	3	6.29 ± 0.27	71.5 ± 4.59	6	6.78 ± 0.54	75.1 ± 8.33	0.195	0.013	0.850	0.777
	45 w	8	6.41 ± 0.30	49.6 ± 8.63	8	6.82 ± 0.43	22.79 ± 7.38	0.032	0.003	0.079	0.004
Isoproterenol+1400W	15 w	4	7.34 ± 0.25	96.4 ± 2.02	7	6.49 ± 0.31	81.8 ± 8.00	0.404	0.980	0.125	0.780
	30 w	8	6.64 ± 0.30	84.0 ± 10.9	6	6.43 ± 0.44	92.3 ± 3.23	0.378	0.418	0.680	0.307
	45 w	8	6.32 ± 0.40	46.52 ± 10.8	8	6.27 ± 0.29	52.7 ± 13.8	0.013	0.002	0.679	0.773

## Data Availability

The data that support the findings of this study are available from the corresponding author upon reasonable request.
